# Highly Sensitive Sensors Based on Metal-Oxide Nanocolumns for Fire Detection

**DOI:** 10.3390/s17020303

**Published:** 2017-02-07

**Authors:** Kwangjae Lee, Young-Seok Shim, Young Geun Song, Soo Deok Han, Youn-Sung Lee, Chong-Yun Kang

**Affiliations:** 1 Contents Convergence Research Center, Korea Electronics Technology Institute (KETI), 11, World cup buk-ro 54-gil, Seoul 03924, Korea; begleam@keti.re.kr; 2 Center for Electronic Materials, Korea Institute of Science and Technology (KIST), 5, Hwarang-ro 14-gil, Seongbuk-gu, Seoul 136-791, Korea; ysshim@kist.re.kr (Y.-S.S.); songyg6397@kist.re.kr (Y.G.S.); 113341@kist.re.kr (S.D.H.); 3 KU-KIST Graduate School of Converging Science and Technology, Korea University, 145, Anam-ro, Seongbuk-gu, Seoul 136-701, Korea

**Keywords:** fire detection, gas sensor, nanostructures

## Abstract

A fire detector is the most important component in a fire alarm system. Herein, we present the feasibility of a highly sensitive and rapid response gas sensor based on metal oxides as a high performance fire detector. The glancing angle deposition (GLAD) technique is used to make the highly porous structure such as nanocolumns (NCs) of various metal oxides for enhancing the gas-sensing performance. To measure the fire detection, the interface circuitry for our sensors (NiO, SnO_2_, WO_3_ and In_2_O_3_ NCs) is designed. When all the sensors with various metal-oxide NCs are exposed to fire environment, they entirely react with the target gases emitted from Poly(vinyl chlorides) (PVC) decomposed at high temperature. Before the emission of smoke from the PVC (a hot-plate temperature of 200 °C), the resistances of the metal-oxide NCs are abruptly changed and SnO_2_ NCs show the highest response of 2.1. However, a commercial smoke detector did not inform any warning. Interestingly, although the NiO NCs are a *p*-type semiconductor, they show the highest response of 577.1 after the emission of smoke from the PVC (a hot-plate temperature of 350 °C). The response time of SnO_2_ NCs is much faster than that of a commercial smoke detector at the hot-plate temperature of 350 °C. In addition, we investigated the selectivity of our sensors by analyzing the responses of all sensors. Our results show the high potential of a gas sensor based on metal-oxide NCs for early fire detection.

## 1. Introduction

A fire alarm system is a device to sense one or more products or phenomena resulting from fire and to immediately warn people through visual and audio appliances [1,2]. One of the most important components of the fire alarm system is the fire detector because it determines whether a fire occurred or not. Generally, detectors for heat, smoke, flame, and gas have been widely used as fire detectors. In particular, gas detectors that sense only specific gaseous compounds such as NO, H_2_, CO, CO_2_, HCl, HCN and volatile organic compounds (VOCs) have attracted great attention since the inhalation of toxic smoke is the primary cause of death from fires [3,4].

Polyvinyl chlorides (PVC) is the world’s third-most widely produced synthetic plastic polymer and it is used in a variety of household products such as packaging, electrical insulation, and interior furnishings [5,6,7,8]. Despite its usefulness, the rapid decomposition with the emission of toxic gases makes PVC the most dangerous material in a fire environment [9]. Among the thermal decomposition products of PVC, HCl, CO and VOCs are the major toxicants as sensory and pulmonary irritants which cause respiratory difficulties or death in the case of inhalation for a long time [10,11]. In addition, since these gases begin to be gradually released at relatively low temperatures below 200 °C, it is possible to extinguish the fire and escape from the fire by detecting HCl, CO, and VOCs in the initial fire stage. Hence, various gas sensors, including photoelectric and ionization sensors for detecting HCl, CO, and VOCs, have been developed [12,13,14,15,16]. However, there are still problems to solve, including mass production, small size, high response and fast response time, and low detection limit.

Recently, chemoresistive gas sensors based on metal oxides are becoming strong candidates for high performance gas detectors due to their extraordinary advantages in simple fabrication methods with high sensitivity and selectivity. The gas-sensing mechanism of the chemoresistive gas sensor is based on electrical properties, including the resistance, current or voltage change of the sensing layer. They are induced from the adsorption and desorption of the gases when specific gases interact with its surface, which is affected by three basic factors, namely the transducer function, utility factor, and receptor function [17,18,19]. For these reasons, various chemoresistive gas sensors with large surface-area-to-volume ratios such as nanotubes [20,21,22], nanobamboos [23], nanowalls [24], nanospheres [25,26], and nanocolumns [27] have been studied based on the three basic factors to enhance the gas-sensing properties. Further, it is well known that highly ordered one-dimensional nanostructures, which show extremely large surface-to-volume ratios, are the most promising material platform for a good gas-sensing performance due to the excellent accessibility of target gases and aggregation-free geometry [19]. Accordingly, our previous studies have investigated the gas-sensing properties of metal-oxide gas sensors based on one-dimensional nanostructures, and already demonstrated their high sensing performance in response to various gases, including C_2_H_5_OH, NO_2_, SO_2_, H_2_, C_7_H_8_, CH_4_, CH_3_COCH_3_, C_6_H_6_, NH_3_, H_2_S, and C_6_H_6_ compared with plain films [23,27,28,29]. However, despite their decent gas-sensing properties, the selectivity of gas sensors based on metal oxides has remained challenging.

In this paper, in order to identify the feasibility of metal-oxide gas sensors as a fire detector and to improve the gas-sensing properties such as sensitivity and selectivity, we fabricated multiple sensors composed of NiO, WO_3_, SnO_2_ and In_2_O_3_ nanocolumns (NCs). GLAD using an electron beam evaporator which is a facile and effective method for mass production is employed to synthesize the highly ordered NiO, WO_3_, SnO_2_ and In_2_O_3_ NCs. PVC is used as a fire source and placed on a hot-plate with elevated temperatures to investigate the gas-sensing properties of our sensors. When the hot-plate temperature reaches 200 °C, the PVC is decomposed and emits product gases. The SnO_2_ NCs firstly responded to the gases, and their response time was much faster than that of a commercial smoke detector for fire detection. At the further elevated temperature of 350 °C, NiO NCs showed the highest response compared with other metal-oxide NCs.

## 2. Experimental Procedure

### 2.1. Fabrication of Sensors

**Figure 1:**
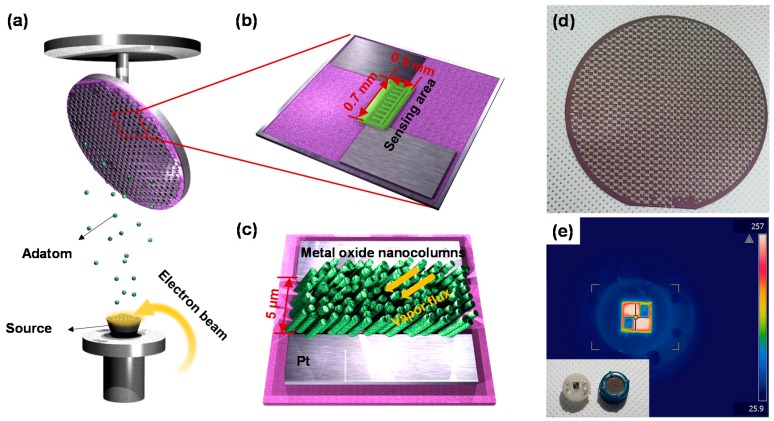
Schematics of (**a**) the wafer-scale fabrication procedure for highly sensitive gas sensors based on metal-oxide NCs; (**b**) a design of Pt IDEs with sensing area; and (**c**) metal-oxide NCs grown I the direction of the vapor flux; (**d**) Photograph of 4 in wafer fully covered with Pt-IDEs patterns; (**e**) Thermographic images showing temperature variation in the Pt-IDEs-patterned substrate at 4 V. Inset shows photograph of our sensor on micro-heater and its cap.

It is well known that GLAD method leads to a variety of columnar nanostructures such as nanorods, nanozigzags, and nanohelixes by controlling the angle of vapor flux with the self-shadowing region [30,31,32]. In order to make the gas sensors based on metal-oxide NCs, we used as the electron beam evaporator, as shown in Figure 1a. Before depositing the sensing films, Pt/Ti (150 nm/30 nm thick) interdigitated electrodes (IDEs) were fabricated on a SiO_2_/Si substrate (1 μm/550 μm thick) using photolithography (followed by an etching procedure) and were cleaned in acetone and ethanol followed by drying in nitrogen gas. Figure 1b,c shows the design of fabricated sample and distances between the Pt/Ti IDEs that are commonly used for sensor’s electrodes to obtain reliable electrical signal (current, resistance, and voltage) are approximately 5 μm with the IDEs area of 0.3 mm × 0.7 mm. The number of sensors on a 4 in wafer is 732, which shows that our fabrication method is possible for mass production, as shown in Figure 1d. For measuring the gas-sensing properties, metal-oxide NCs deposited 4 in SiO_2_/Si was precisely cut by a dicing saw and placed on the Pt heater-patterned alumina substrate. Figure 1e shows the thermographic image of Pt heater and the temperature of Pt heater reached approximately 250 °C at an applied bias of 5 V.

### 2.2. Characterization of Metal-Oxide NCs

**Figure 2:**
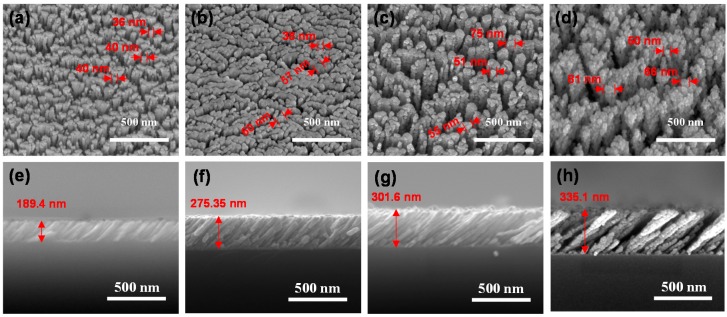
Plain-view SEM images of the (**a**) NiO; (**b**) WO_3_; (**c**) SnO_2_ and (**d**) In_2_O_3_ NCs; (**e**–**h**) show cross-sectional SEM images of (**a**–**d**), respectively.

The density of the metal-oxide NCs influences the accessibility of the target gases. In order to form the porous structures, we fixed the angle of the substrate at 80° and deposited all the films. The substrate was located 30 cm away from the crucible and shadow masks were used to deposit the films only on the IDEs patterns. The base pressure and growth rates were 5 × 10^−6^ Torr and 1 Å·s^−1^, respectively. The morphologies of the fabricated metal-oxide NCs after annealed at 550 °C for 2 h were observed by a field emission scanning electron microscope (FESEM: SU-70, Hitachi) using an acceleration voltage of 15 kV and a working distance of 10 mm. Figure 2a–f show the top-view and cross-sectional FE-SEM images of all samples, respectively. The thicknesses of metal-oxide NCs are controlled by thickness monitor based on Quartz crystal (6 MHz Gold). Although we put the exact literature value into material density and acoustic impedance, there occurs the deviation of thickness. However, metal-oxide NCs with porous structure are successfully formed. In order to measure the thickness of metal-oxide NCs, we used the measurement tool in the SEM system. Interestingly, despite same deposition angle, thicknesses of the NiO, WO_3_, SnO_2_ and In_2_O_3_ NCs are 189.4, 272.44, 301.6 and 335.1 nm, respectively. Also, diameters of the NiO, WO_3_, SnO_2_ and In_2_O_3_ NCs were measured to be ~40 nm, ~57 nm, ~63 nm and ~66 nm, respectively, which can be attributed different diffusion coefficients of adatoms depending on materials [30,31].

**Figure 3:**
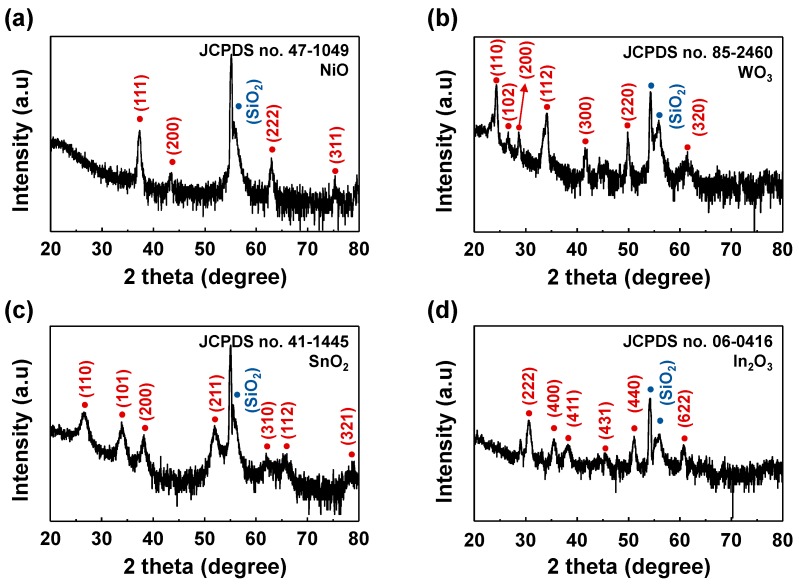
X-ray diffraction pattern of the (**a**) NiO; (**b**) WO_3_; (**c**) SnO_2_ and (**d**) In_2_O_3_.

The NCs were characterized by X-ray Diffraction (XRD: DMax2500, Rigaku, Japan) films with 2θ scan from 20° to 80°, where CuKα radiation (wavelength = 1.5418 Å) was used for the X-ray source and the fixed incident angle of 2°. The diffraction peaks indexed as NiO (JCPDS no. 47-1049), WO_3_ (JCPDS no. 85-2460), SnO_2_ (JCPDS no. 41-1445), In_2_O_3_ (JCPDS no. 06-0416) and substrate (SiO_2_/Si) indicate that all the nanocolumns are polycrystalline. From the XRD results, no remarkable difference in crystallinity is observed, as presented in Figure 3a–c.

### 2.3. Gas-Sensing Measurement of Metal-Oxide NCs and Fire Detection Method

**Figure 4:**
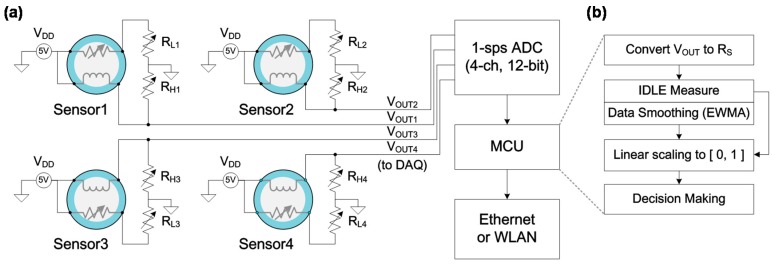
(**a**) Interface circuitry and block diagram of the fire detection module with metal-oxide gas sensors; (**b**) flow chart of the decision process.

A diagram and a flow chart of the fire detection module are shown in Figure 4. The module contains analog signal conditioning circuitry which is composed of four different gas sensors, their potentiometric circuits, a 12-bit analog-to-digital converter (ADC), a microcontroller unit (MCU) and a communication unit such as Ethernet or wireless local area network (WLAN). The signal conditioning circuitry plays a role of data acquisition in measuring the output of sensors at 1 s intervals for further processing. The MCU performs pre-signal processing and makes a decision on whether a fire occurred or not. The fire detection module is a sensor node which integrates sensors and a communication unit. This is a base unit of surveillance sensor network system for the whole house. For the fire decision, the measured sensor data are preprocessed using a low pass filter and a normalization operation, and then decision making is performed as described in Figure 4b. The interface circuitry measures voltage level of each sensor to obtain changes of the sensor resistance ($R_{S}$). This typically consists of a simple voltage divider. Because the measured magnitude is the output voltage ($V_{OUT}\left[i\right]$), we can easily transform the metal-oxide resistance changes $R_{S}\left[i\right]$ by solving Equation (1).

(1) $$R_{S}\left[i\right]=\frac{R_{L}\times \left(V_{DD}-V_{OUT}\left[i\right]\right)}{V_{OUT}\left[i\right]},$$

where $V_{DD}$ is the circuit voltage and $R_{L}$ is the load resistance. The obtained data have temporal noise due to the electronics of the detection module. To suppress noise, the sensor data are filtered using an Exponential Weighted Moving Average (EWMA) filter [33]. This filter is suitable for time series data and the data can be calculated recursively using the following equations:

(2) s[0]=x[0]s[n]=α⋅s[n−1]+(1−α)⋅x[n], ∀n≥1,

where x[0], x[n] is the sensor resistance, s[0], …, s[n] is the filtered sensor resistance and α is a smoothing factor. The factor has to set a value between 0 and 1. Values which are close to 1 results in an aggressive smoothing while values which are close to 0 nearly preserve the original data. The metal-oxide gas sensors have a different dynamic range due to differences in the sensor material and structure. When highly sensitive sensors respond to target gases, their resistance drops to 1/10,000 from 1/1000 compared to their resistance in air ambient. Thus, before decision making process, we convert the dynamic ranges of the sensors comparable by unity-based normalized data of each sensor to the interval [0,1] using the following linear transformation [34,35]:

(3) $$s^{\prime }\left[i\right]=\frac{s\left[i\right]-S_{min}}{S_{max}-S_{min}},\;0\leq i\leq n,$$

where $s^{\prime }\left[i\right]$ is the normalized sensor response and $S_{min}$, $S_{max}$ are minimum, maximum value of filtered data among s[0], …, s[n], respectively. In most cases, $S_{max}$ indicates baseline sensor resistance where sensor is in air ambient and $S_{min}$ indicates sensor resistance exposed to maximum concentration of toxic gases. However, in a fire accident, the sensor module does not know the maximum concentration of generated gases because the concentration of the gases varies on various conditions such as burning material, building structure, etc. And as explained before, $S_{min}$ is very small value compared to $S_{max}$. So $S_{min}$ has minor influence in Equation (3). Thus, the equation is approximated to the following equations:

(4) $$s\text{′}\left[i\right]=\frac{s\left[i\right]}{S_{max}}=\frac{s\left[i\right]}{\frac{1}{N}\cdot \sum_{k=HeaterOn-Nsec}^{HeaterOn}s\left[k\right]},\;\forall i\geq N.$$

This equation also transforms data of other type sensors to normalized data by linear scaling. That means that performance evaluation with other sensors is possible. A decision making is a process of determining the fire using the change of the signal. The decision making of this module uses a simple threshold decision method. Although there are many sophisticated decision methods, they use the characteristics of a sensor itself to improve their performance. Thus, instead of these methods, the threshold decision method is used for decision making as a general performance evaluation of sensors.

## 3. Results and Discussion

### 3.1. Gas Response of Metal-Oxide NCs as a Function of the Hot-Plate Temperature

**Figure 5:**
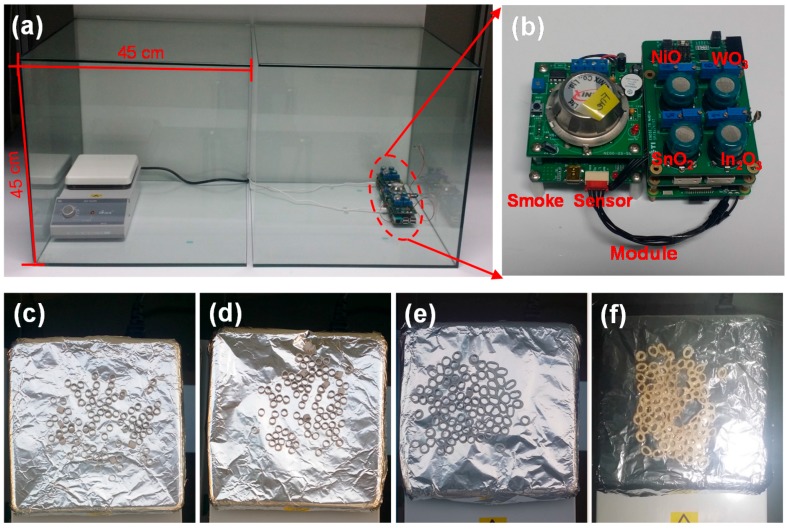
Photographs of test environment and results; (**a**) a chamber for gas-sensing measurement and (**b**) the signal processing circuits with the integrated sensors; (**c**–**f**) PVC deformation at varying hot-plate temperatures (50 °C, 100 °C, 200 °C and 350 °C).

**Figure 6:**
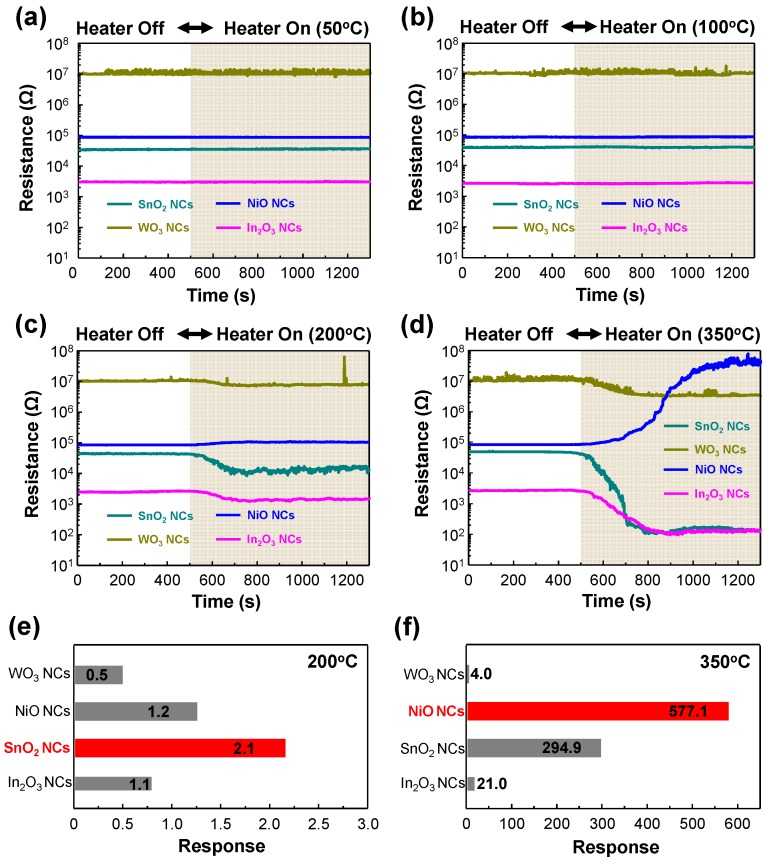
Real-time transient response of all samples (SnO_2_, NiO, WO_3_ and In_2_O_3_) at different hot-plate temperatures: (**a**) 50 °C, (**b**) 100 °C, (**c**) 200 °C and (**d**) 350 °C. Responses of all samples at hot-plate temperatures of (**e**) 200 °C and (**f**) 350 °C.

In order to investigate the gas-sensing properties of our sensors, we prepared a hot-plate with 5 g PVC and a sensor module in glass boxes (90 cm × 45 cm × 45 cm), as shown in Figure 5a,b. Generally, the gas-sensing properties of metal oxides are affected by the amount of ionized oxygen species (O_2_^−^, O^−^, O^2−^) on the surface, and we fixed the operating temperature of our sensors at approximately 250 °C under consideration for the adsorption of oxygen species and power consumption, as shown in Figure 1e [36]. Firstly, we increased the hot-plate temperature to observe the decomposition of the PVC. Figure 5c–f shows the photographs of PVC deformation at different hot-plate temperatures. For hot-plate temperatures of 50 °C and 100 °C, there was no change in the PVC. However, when the hot-plate temperature reached 200 °C, the PVC started to melt and it emitted smoke at a hot-plate temperature at 350 °C. Figure 6a–d shows the real-time response curves as a function of the hot-plate temperatures. To obtain thermal stability, all samples were aged for 30 min after each sensing measurement. Upon exposure to air ambient (off-state of hot-plate), the base resistances of SnO_2_, NiO, WO_3,_ and In_2_O_3_ were approximately 3 × 10^4^, 1 × 10^5^, 1 × 10^7^ and 3 × 10^3^ Ω, respectively. There were no resistance changes in all samples at hot-plate temperatures of 50 °C and 100 °C. However, when the PVC was exposed to a hot-plate temperature of 200 °C, the resistances of samples were slightly changed, which indicates that the PVC emitted gases such as HCl, CO, VOCs and various compounds due to decomposition by applied heat. In addition, the shape of the response curves shows that SnO_2_, WO_3,_ and In_2_O_3_ are *n*-type semiconductors and NiO is a *p*-type semiconductor, respectively, which means the reducing gases such as CO and VOCs were mainly emitted from the PVC. Upon exposure to a hot-plate temperature of 350 °C, the resistance changes of the samples were gradually increased and saturated. Figure 6e,f shows the responses of each sample at hot-plate temperatures of 200 °C and 350 °C. The response is defined as *R*_HF_/*R*_HO_ for *n*-type semiconductors and *R*_HO_/*R*_HF_ for the *p*-type semiconductor, where *R*_HF_ and *R*_HO_ denote resistances in the off-state and on-state of the hot-plate, respectively. The responses of NiO, WO_3,_ In_2_O_3_ and SnO_2_ were 1.2, 0.5, 2.1 and 1.1 at the hot-plate temperature of 200 °C, respectively. At the hot-plate temperature of 350 °C, the responses of NiO, WO_3,_ In_2_O_3_ and SnO_2_ were 557.1, 4.0, 21.0 and 294.9, respectively.

We infer that these results are attributed to the detection ranges and the selectivity to the target gases of all the materials [17]. Firstly, the correlation between the response and the gas concentration can be understood with the Langmuir adsorption isotherm theory of dissociated gas [37]. When emitted gas molecules react with pre-absorbed oxygen, the variation of the resistance changes might be proportional to the fraction (θ) of the active site that is covered by the pre-absorbed oxygen and linearly affects the effective carrier concentration in the sensing material. Therefore, the resistance change of a sensor is proportional to θ. The adsorption rate of emitted gases is $\kappa_{1}p{\left(1-\theta \right)}^{2}$ and the desorption rate is $\kappa_{2}\theta^{2}$, where $\kappa_{1}$ and $\kappa_{2}$ are the adsorption and desorption constant, respectively, and p represents the partial pressure of the emitted gases from the PVC. When the resistance reaches equilibrium, the adsorption rate equals the desorption rate:

(5) $$\kappa_{1}p{\left(1-\theta \right)}^{2}=\kappa_{2}\theta^{2}\;,\mathrm{or}$$

(6) $$\theta /\left(1-\theta \right)={\left(\kappa_{1}/\kappa_{2}\right)}^{1/2}p^{1/2}$$

At low coverage of the emitted gases (θ≪1), the resistance changes, is expected to be proportional to the square root of the emitted gas concentration, and eventually becomes partially saturated when θ is close to 1. Thus, the response cannot be highly enhanced for partial saturation state as increase the emitted gas concentration.

(7) $$\Delta R/R_{0}\propto \theta \approx {\left(\kappa_{1}/\kappa_{2}\right)}^{1/2}p^{1/2}$$

In this case, the ${\left(\kappa_{1}/\kappa_{2}\right)}^{1/2}$ value determines the slope of the response. Since many other papers show that SnO_2_ has a superior sensing property to NiO, we can infer that [38,39,40]:

(8) $${\left[{\left(\kappa_{1}/\kappa_{2}\right)}^{1/2}\right]}_{SnO_{2}}>{\left[{\left(\kappa_{1}/\kappa_{2}\right)}^{1/2}\right]}_{NiO}$$

From the results, we can explain that SnO_2_ NCs were saturated earlier than NiO, and had an enhanced adsorption and catalytic capability towards the emitted gases from the PVC. Therefore, as the gas concentration increased, it seems that the active areas of the SnO_2_ surface were partially saturated. In contrast, the response of NiO NCs showed a constant increase up to 350 °C. Hence, at the hot-plate temperature of 350 °C, NiO NCs showed the highest response value.

Secondly, it is well-known that the response of *p*-type semiconductors is as low as the square root of that of *n*-type semiconductors when structural factors are equal, and a low response can yield to poor selectivity of various gases [41]. Interestingly, the response of NiO NCs at the hot-plate temperature of 200 °C was higher than that of WO_3_ and In_2_O_3_ NCs. Furthermore, NiO NCs exhibited the highest response at the hot-plate temperature of 350 °C. To the best of our knowledge, there are no reports that the responses of sensors based on *p*-type metal oxides are higher than those of sensors based on *n*-type metal oxides. From the results, we carefully inferred that the PVC emits the reducing gases which dominantly react with NiO NCs.

### 3.2. Early Fire Detection of Metal-Oxide NC–Based Gas Sensor

**Figure 7:**
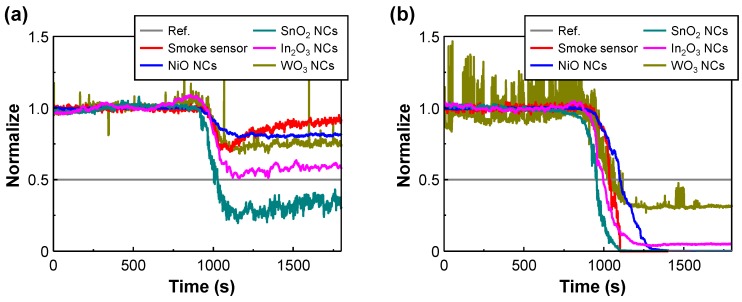
Normalized transient response curves of all samples (SnO_2_, NiO, WO_3_ and In_2_O_3_) and commercial sensor at different hot-plate temperatures: (**a**) 200 °C and (**b**) 350 °C.

Figure 7 shows the normalized transient response curves of the metal-oxide gas sensors and a commercial smoke detector (NIS-05A, NEMOTO) at different hot-plate temperatures. The threshold level (Ref.) was set to 0.5, represented by a gray line. Upon exposure to air ambient, the baselines of SnO_2_, NiO, WO_3_, In_2_O_3_ NCs and the smoke sensor were approximately 1. When the PVC was exposed to a hot-plate temperature of 200 °C at a time transient of 750 s, then the normalized transient response of the samples and the smoke sensor changed, as shown in Figure 7a. The response of SnO_2_ NCs met the threshold at 1007 s, while other responses did not meet the threshold. When the PVC was exposed to a hot-plate temperature of 350 °C at a time transient of 725 s, then the normalized transient response of the samples and the smoke sensor drastically changed, as shown in Figure 7b. The response of SnO_2_, NiO, WO_3_, In_2_O_3_ NCs and the smoke sensor met the threshold at 948, 1105, 1029, 997 and 1039 s, respectively. The metal-oxide gas sensors, except the NiO NCs, reacted faster than the smoke sensor. Further, the difference in the transient response between the SnO_2_ NCs and the smoke sensor from temperature elevation was 91 s. These results also show that the transient response of the NiO NCs continued their reaction to the gases until saturation at approximately 1270 s, which means that the fire can be more quickly detected by using SnO_2_ NCs than the smoke sensor, and the fire detection module using the NiO NCs can measure a higher concentration of generated gas than the others since they can maintain their reaction for a longer time.

## 4. Conclusions

Highly ordered metal-oxide NCs were fabricated using a facile and effective method based on GLAD. Upon exposure to a fire ambient, their sensing behaviors were significantly different depending on the sensing materials. All sensors started to react with the gases emitted from the PVC at a hot-plate temperature of 200 °C. The response of SnO_2_ NCs was 2.1, which was the highest response among the four different sensors at 200 °C. At the hot-plate temperature of 350 °C, the PVC emitted smoke and the response of all samples dramatically increased. Interestingly, the response of NiO NCs was two times greater than that of SnO_2_ NCs. Moreover, the response time of SnO_2_ NCs was much faster than that of the smoke sensor at the hot-plate temperature of 350 °C. Although we did not know the exact concentrations and types of gases emitted from the PVC, these results indicate that NiO NCs are more effective for detecting the high concentration of the gases emitted from the PVC at high temperatures and SnO_2_ NCs are able to detect fire early. The excellent gas-sensing properties of metal-oxide NCs could be explained by two effects. Firstly, the large surface-to-volume ratios of metal-oxide NCs enhanced the reaction site of the target gases, leading to a high response. Secondly, the optimal density of metal-oxide NCs improved the accessibility of the target gases, which is related to the response time. Consequently, we believe that our sensors are very promising for early fire detection or detection at various stages of fire. In the future, if multi-sensors sensitive to other fire factors are applied to the proposed system, it can immediately respond to diverse fire risks.
